# Salol in Typhoid Fever

**Published:** 1891-10-03

**Authors:** 


### THERAPEUTICS.

### SALOL IN TYPHOID FEVER.

We have recently noted the prevailing tendency to treat enteric fever as a microbal disease, and one which lends itself from the situation of the chief lesions to at least a modification in a desirable direction of the dangerous features which characterise this disease. Salol is an intestinal antiseptic which appears to fulfil most of the conditions essential for success. It is a salicylate of phenol discovered by M. V. Necke, is insoluble in water, and quite tasteless. In 1886 it was introduced by M. Sahli, and was then stated to be insoluble in the stomach, but quickly decomposing when it comes in contact with the pancreatic fluid, and breaking up into salicylic acid and phenol in the intestines. Its value in acute and chronic rheumatism is well known. Although it was for a long time doubtful whether the above-mentioned reaction and splitting up of salol really did take place in the intestines, there is very little doubt on this point after the experiments of Papuli (*Rivista clinica*) but it was further shown by this observer that the reaction was brought about by micro-organisms. Most of the many forms of bacteria possess this property of decomposing salol, and the more numerous they are the greater activity they display in decomposing the acid, and germs which do not induce the separation of the component acid and phenol are weakened or destroyed by the presence of phenol and salicylic acid resulting from the action of other microbes present. Dr. W. C. Cahall, having made trial of salol in every case of typhoid fever coming under his care during the past two years, speaks very highly of its effects. The dose employed should be at least three grains, given every two hours, day and night, in the form of powder. Being tasteless, it is readily and easily taken. Under its influence he found tympanites disappear; and diarrhœa, no matter how severe at the commencement, progressively improves, and requires no further special treatment. According to Dr. Cahall, the temperature seldom rose after the first day of treatment, and after the first week it steadily and more or less rapidly fell. The pulse did not show the usual increase in frequency, but remained full, and seldom exceeded one hundred; in none of the cases was the action of the heart such as to call for alcohol. The only untoward effect observed was a partial suppression of urine in some cases, but in no instance was it necessary to suspend the treatment on this account. The average duration of sixteen cases treated by this method was seventeen days, and the only complication met with was pneumonia, which was present in two cases.* The action of phenol on the kidneys has always rendered dubious the advisability of giving anything like large doses in enteric fever, but recent investigations have shown that salol is only very gradually split up into its constituents in the intestines.

* *Medical News*, November, 1890; *Pract.*, January, 1891.

